# Supplementary material for: Mitochondrial RNA processing in absence of tRNA punctuations in octocorals
Source: BMC Mol Biol. 2017 Jun 17;18:16. doi: 10.1186/s12867-017-0093-0 (PMC5474008; doi:10.1186/s12867-017-0093-0)
Supplement: Supplementary file 1 — Additional file 1. Mitochondrial genome of Sinularia cf. cruciata. [file 12867_2017_93_MOESM1_ESM.doc]

**Additional file 1: Mitochondrial genome of *Sinularia* cf. *cruciata.***

**Methods**

The complete mitochondrial genome of *Sinularia* cf. *cruciata* was obtained following standard procedures, which included PCR amplification of overlapped fragments (up to 3 kb) and Sanger sequencing. Mitochondrial DNA fragments were amplified using previously published primers sets and PCR protocols [1-3]. Amplicons of different sizes (up to 3kb) were purified to remove unincorporated primers and were directly sequenced using BigDye® Terminator v3.1 chemistries on an ABI 3730 DNA Analyzer (Applied Biosystems, Foster City, CA, USA). Sequences quality was assessed by visually checking the chromatograms. Mitogenome was assembled using *Sinularia peculiaris* (Accession number: JX023274) as reference in Geneious 8.1.7 [4]. A preliminary screening of the coding-protein regions was carried using the “ORFs finder” tool implemented in Geneious and the resulting annotations were further compared by similarity with those of other octocoral mitogenomes. Detection of control region repeats and tRNAs was performed using Tandem Repeats Finder [5] and tRNA-scan SE [6], respectively. Nucleotide diversity (π) between *Sinularia* cf. *cruciata* and *Sinularia peculiaris* was calculated in DnaSP 5 [7] using a sliding window of 500 bp (25 bp steps). For this analysis neither nucleotide ambiguities nor sites with gaps were considered. A graphical map including comparative mitogenomic information between the two *Sinularia* species was inferred with the CGView Server [8].

**Results**

**Table S1**

Mitochondrial genome organisation of *Sinularia* cf. *cruciata*.

| **Species** | **Gene** | **Feature** | **Position** | | **Codon** | | **Strand** | **Lenght (bp)** | **Intergenic Region** |
| --- | --- | --- | --- | --- | --- | --- | --- | --- | --- |
|  |  |  | Start | Stop | Start | Stop |  |  |  |
| *Sinularia* cf. *cruciata* | *COI* | CDS | 1 | 1590 | ATG | TA | H | 1582 | 8 |
|  | 12S | rRNA | 1749 | 2673 |  |  | H | 925 | 4 |
|  | *ND1* | CDS | 2678 | 3649 | ATG | TAG | H | 972 | 27 |
|  | *CytB* | CDS | 3677 | 4843 | ATG | TAA | H | 1167 | 30 |
|  | *ND6* | CDS | 4874 | 5431 | ATG | TAG | H | 558 | 44 |
|  | *ND3* | CDS | 5476 | 5829 | ATG | TAG | H | 354 | 19 |
|  | *ND4L* | CDS | 5849 | 6142 | ATG | TAA | H | 294 | 13 |
|  | *mtMutS* | CDS | 6156 | 9137 | ATG | TAA | H | 2982 | 9 |
|  | 16S | rRNA | 9147 | 11114 |  |  | H | 1968 | 31 |
|  | *ND2* | CDS | 11146 | 12519 | ATG | TAG | H | 1374 | -13 |
|  | *ND5* | CDS | 12507 | 14324 | ATG | TAA | H | 1818 | 97 |
|  | *ND4* | CDS | 14422 | 15870 | ATG | TAA | H | 1449 | 56 |
|  | tRNA-Met | tRNA | 15927 | 15997 |  |  | L | 71 | 39 |
|  | *COIII* | CDS | 16037 | 16822 | ATG | TAG | L | 786 | 64 |
|  | *ATP6* | CDS | 16887 | 17594 | ATG | TAA | L | 708 | 24 |
|  | *ATP8* | CDS | 17619 | 17834 | ATG | TAA | L | 216 | 22 |
|  | *COII* | CDS | 17857 | 18618 | ATG | TAA | L | 762 | 112 |

**Figure S1.** Complete mitogenome of *Sinularia* cf. *cruciata* and genomic comparisons with *Sinularia peculiaris*.

**(A)** Graphical view of the mitochondrial genome of *Sinularia* cf. *cruciata* with genome size and gene annotations. GC content is shown in black, GC skew is plotted for the entire sequence in green (GC skew +) and purple (GC skew -). The inner pink ring shows the BLAST hit detected by the *blastn* search against *Sinularia peculiaris* mitogenome.

**(B)** Sliding window analysis of the complete mitogenomes of *Sinularia* cf. *cruciata* and *Sinularia peculiaris*. Nucleotide diversity across the genome is shown by the black line in a window of 500 bp (25 bp steps). Grey panels show the most variable regions across the two *Sinularia* species.

**
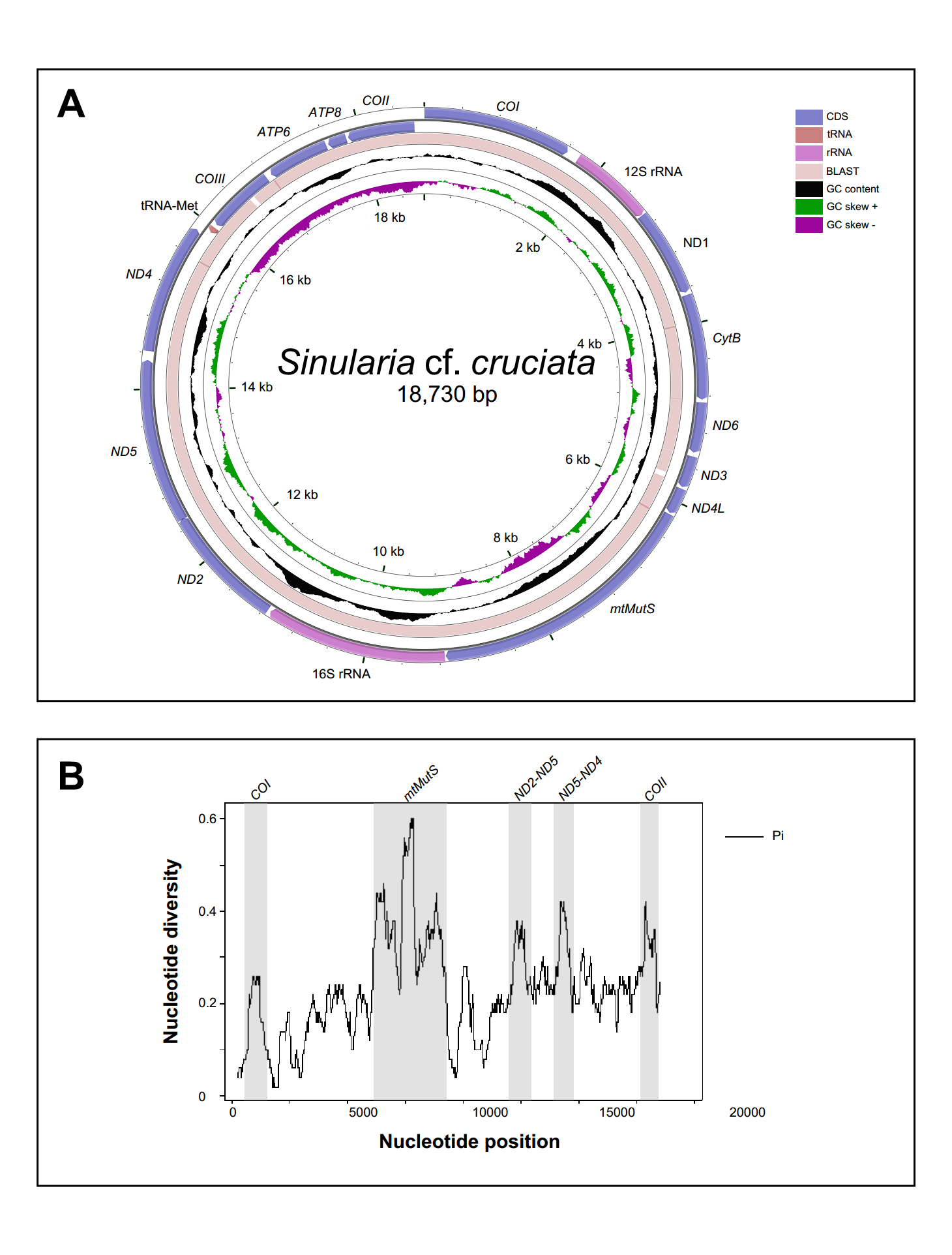
**

**References**

1. Kayal E, Bentlage B, Collins AG, Kayal M, Pirro S, Lavrov DV: **Evolution of linear mitochondrial genomes in medusozoan cnidarians**. *Genome biology and evolution* 2012, **4**:1-12.

2. Brockman SA, McFadden CS: **The mitochondrial genome of Paraminabea aldersladei (Cnidaria: Anthozoa: Octocorallia) supports intramolecular recombination as the primary mechanism of gene rearrangement in octocoral mitochondrial genomes**. *Genome biology and evolution* 2012, **4**:994-1006.

3. Burger G, Lavrov DV, Forget L, Lang BF: **Sequencing complete mitochondrial and plastid genomes**. *Nat Protoc* 2007, **2**(3):603-614.

4. Kearse M, Moir R, Wilson A, Stones-Havas S, Cheung M, Sturrock S, Buxton S, Cooper A, Markowitz S, Duran C *et al*: **Geneious Basic: an integrated and extendable desktop software platform for the organization and analysis of sequence data**. *Bioinformatics* 2012, **28**(12):1647-1649.

5. Benson G: **Tandem repeats finder: a program to analyze DNA sequences**. *Nucleic Acids Res* 1999, **27**(2):573-580.

6. Schattner P, Brooks AN, Lowe TM: **The tRNAscan-SE, snoscan and snoGPS web servers for the detection of tRNAs and snoRNAs**. *Nucleic Acids Res* 2005, **33**(Web Server issue):W686-689.

7. Librado P, Rozas J: **DnaSP v5: a software for comprehensive analysis of DNA polymorphism data**. *Bioinformatics* 2009, **25**(11):1451-1452.

8. Grant JR, Stothard P: **The CGView Server: a comparative genomics tool for circular genomes**. *Nucleic Acids Res* 2008, **36**(Web Server issue):W181-184.
