# Supplementary material for: Mitochondrial RNA processing in absence of tRNA punctuations in octocorals
Source: BMC Mol Biol. 2017 Jun 17;18:16. doi: 10.1186/s12867-017-0093-0 (PMC5474008; doi:10.1186/s12867-017-0093-0)

Additional file 2: Mitochondrial gene expression determined by RNA-Seq for *Sinularia* cf. *cruciata*, *Gorgonia ventalina*, and *Corallium rubrum.*

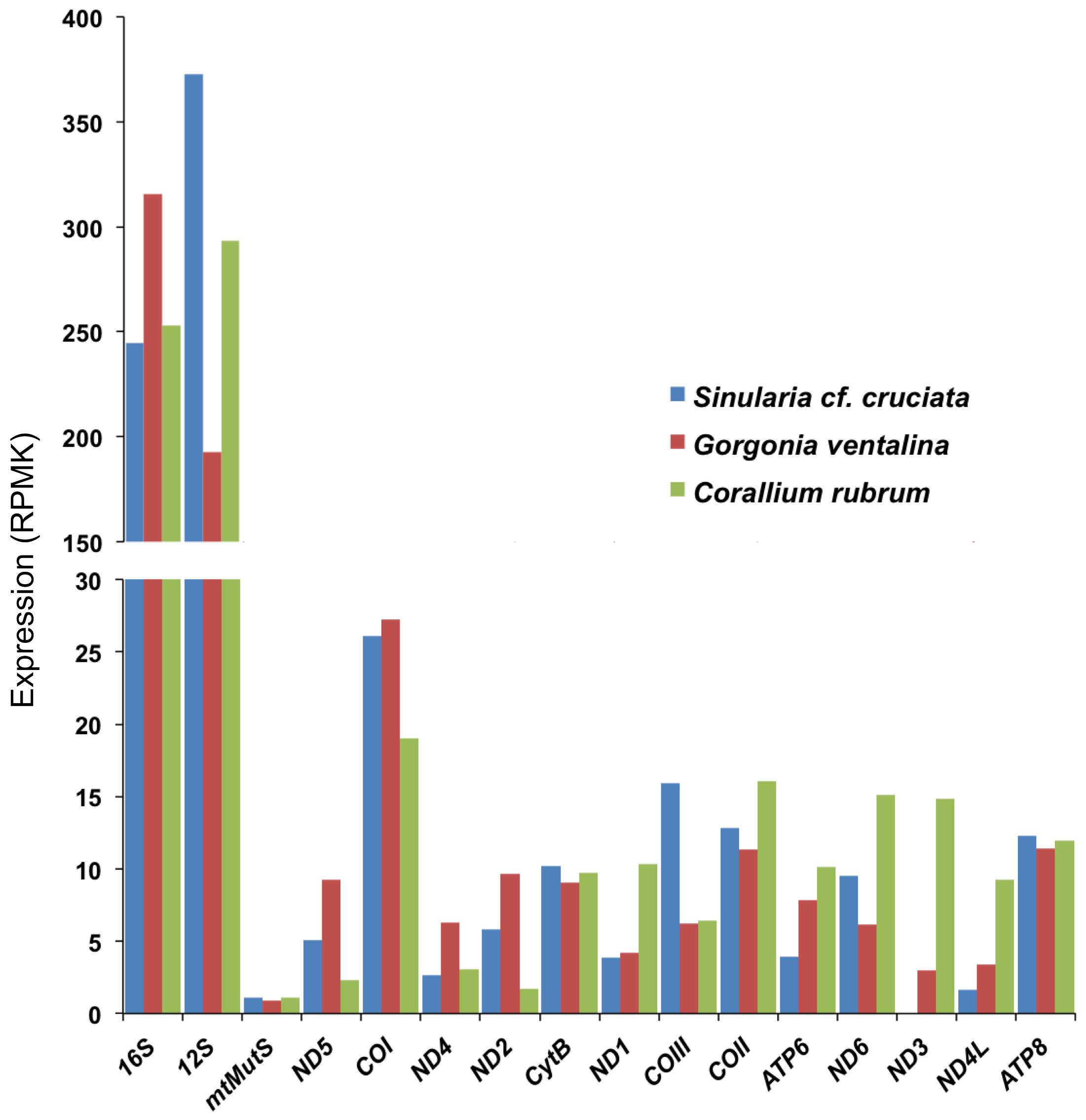

Supplement: Supplementary file 2 — Additional file 2. Mitochondrial gene expression determined by RNA-Seq for Sinularia cf. cruciata, Gorgonia ventalina, and Corallium rubrum. [file 12867_2017_93_MOESM2_ESM.pdf]
