# Supplementary material for: Mitochondrial RNA processing in absence of tRNA punctuations in octocorals
Source: BMC Mol Biol. 2017 Jun 17;18:16. doi: 10.1186/s12867-017-0093-0 (PMC5474008; doi:10.1186/s12867-017-0093-0)

Additional file 3: Stem-loop structures and conserved motifs of putative control regions of octocorals studied.

(A) Predicted stem-loop structures of the four IGRs

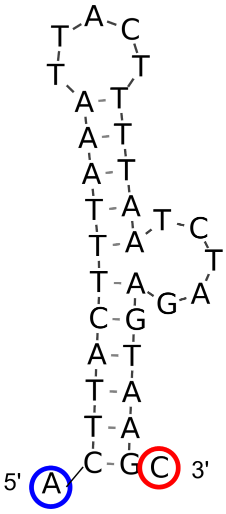

Sinularia* cf. *cruciata
COII-COI IGR

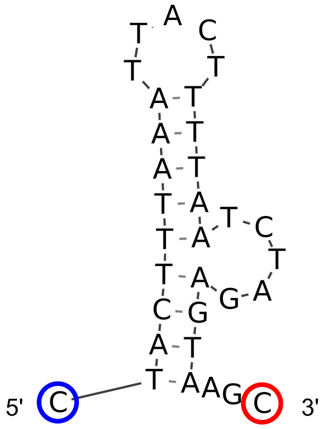

***Sinularia piculiaris* (NC_018379)**
COII-COI IGR

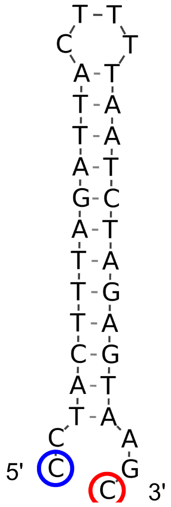

***Pseudopterogorgia bipinnata* (NC_008157)**
COII-COI IGR

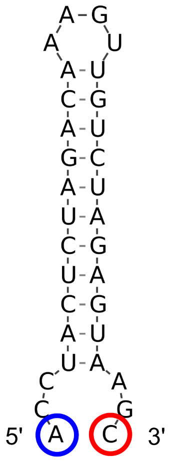

***Corallium rubrum* (NC_022864)**
ND6-COI IGR

(B) Graphical representation of conserved motif of aligned putative control region (3' to 5') from octocoral studied here, generated using GLAM2 from the MEME Suite 4.11.2 (meme-suite.org/)

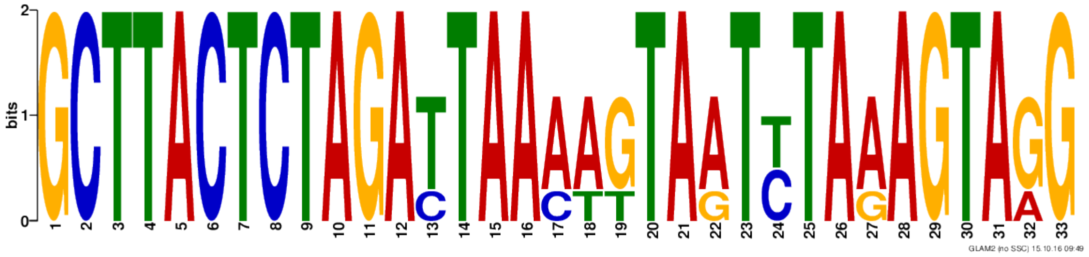

Supplement: Supplementary file 3 — Additional file 3. Stem-loop structures and conserved motifs of putative control regions from octocorals mitogenomes studied. [file 12867_2017_93_MOESM3_ESM.pdf]
