# Supplementary material for: Mitochondrial RNA processing in absence of tRNA punctuations in octocorals
Source: BMC Mol Biol. 2017 Jun 17;18:16. doi: 10.1186/s12867-017-0093-0 (PMC5474008; doi:10.1186/s12867-017-0093-0)
Supplement: Supplementary file 4 — Additional file 4. Additional tables (1, 2). [file 12867_2017_93_MOESM4_ESM.pdf]

## Additional File 4: Additional Tables.

**Table S2:** Positive PCR amplification of cDNA using primers binding different regions of *ND3-ND4L-mtMutS* transcription units

| Sr. No. | Primer Pair Forward/Reverse | Region | Length (bp) |
|---|---|---|---|
| 1 | M-6156/M-9137 | Complete *mtMutS*/Start-Stop codons | 2981 |
| 2 | N4L-6071/M-8826 | *ND4L-mtMutS* | 2755 |
| 3 | N3-5499/M-7199 | *ND3-ND4L-mtMutS* | 1700 |
| 4 | N3-5499/M-6780 | *ND3-ND4L-mtMutS* | 1281 |
| ***** | **Primers** | **Sequence (5'---> 3')** | |
| 1 | M-6156 | ATGAATCAGATACCTATGC | |
| 2 | M-9137 | TTACTCAGTTCCACTGTC | |
| 3 | N4L-6071 | GCCATTATGGTTAACTATTAC | |
| 4 | M-8826 | CACTTCGGGATGGTAACTCC | |
| 5 | N3-5499 | ACTACTTATCGTCAGCGGAAC | |
| 6 | M-7199 | AGGCAATAAGTCCAATTGATATTCTGCTCG | |
| 7 | M-6780 | TTAAGCCAACCCCCGAGTCC | |

Primer annotations as per *S.* cf. *cruciata* mitogenome (KY462727).

**Table S3:** qPCR primers used for quantification of alternative transcripts.

| No. | Gene | Nt. Position | Primer Sequences (5' to 3') | Amplicon Size (bp) |
|---|---|---|---|---|
| **A.** | **Reference gene primers** | | | |
| 1 | *ACTB* | β-Actin* | for: CCAAGAGCTGTGTTCCCTTC<br>rev: CTTTTGCTCTGGGCTTCGT | 107 |
| **B.** | **The *mtMutS* gene primers** | | | |
| 1 | *mtMutS*-A1 | 6263-6381 | for: GCATGAGCCCGATACTTCTAGT<br>rev: ACGAAGCAACTTGTTCAATGG | 118 |
| 2 | *mtMutS*-A2 | 6263-6719 | for: GCATGAGCCCGATACTTCTAGT<br>rev: CCGGGTTACTTTGTCCCTGTCCG | 456 |
| 3 | *mtMutS*-B1 | 6655-6940 | for: CAGCCATGAATGGGCATAG<br>rev: TSGAGCAAAAGCCACTCC | 285 |
| 4 | *mtMutS*-B2 | 6655-6780 | for: CAGCCATGAATGGGCATAG<br>rev: TTAAACCTACCCCCGAGTCC | 125 |
| 5 | *mtMutS*-C1 | 9002-9083 | for: GGTGCCAGTTTGTTCAAGC<br>rev: ATGTCCTGGGGTTCTCTTCC | 81 |
| 6 | *mtMutS*-C2 | 9002-9137 | for: GGTGCCAGTTTGTTCAAGC<br>rev: TTACTCAGTTCCACTGTC | 135 |

* Gene name
Nucleotide positions as per *Sinularia* cf. *cruciata* mitogenome (Acc. No. KY462727)
