# Supplementary material for: Mitochondrial RNA processing in absence of tRNA punctuations in octocorals
Source: BMC Mol Biol. 2017 Jun 17;18:16. doi: 10.1186/s12867-017-0093-0 (PMC5474008; doi:10.1186/s12867-017-0093-0)
Supplement: Supplementary file 5 — Additional file 5. RHAPA analysis. [file 12867_2017_93_MOESM5_ESM.pdf]

Additional File 5: RHAPA Analysis

(A) Gel image showing successful RNase H-assisted cleavage of distal 3'end of *mtMutS* transcript. It was followed by oligo(dT)-cDNA synthesis and PCR with different primer pairs depicted in (B) to verify the success of RHAPA assay. Lack of amplification in PCR with primer pair used in "d" indicated success. Positive amplification in "a'', "b" and "c" using cDNA template prepared after cleavage suggest presence of alternate transcripts for *mtMutS* gene.

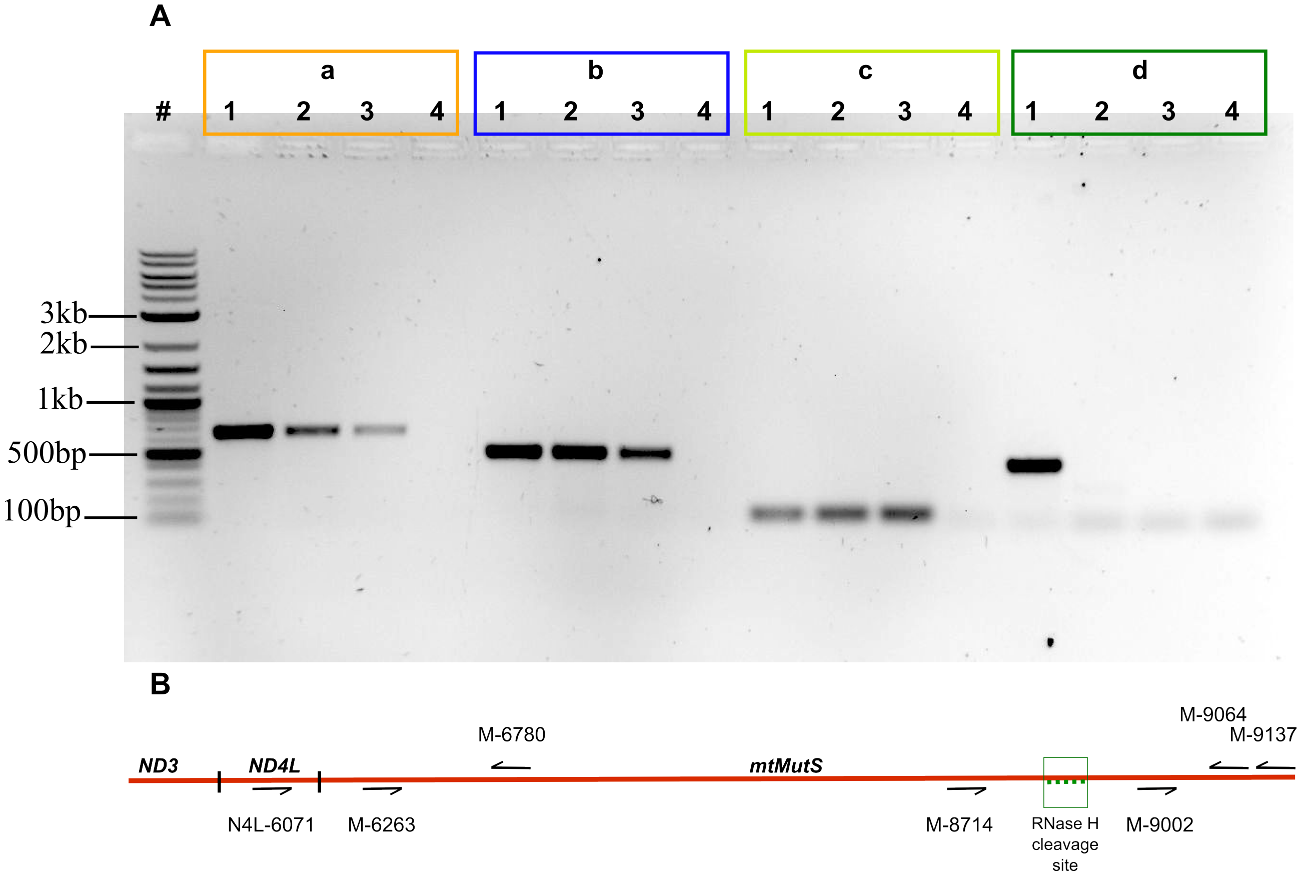

Gel details:

# = 2-Log DNA Ladder (NEB)
a = N4L-6071 + M-6780
b = M-6263 + M-6780
c = M-9002 + M-9137
d = M-8714 + M-9064 (5'ATGTCCTGGGGTTCTCTTCC 3')
1 = Positive control (*S*. cf. *cruciata* DNA)
2 = *S*. cf. *cruciata* cDNA
3 = *Sinularia sp.* cDNA
4 = Negative control

Note: For primer sequences refer to the tables provided.
