# Supplementary material for: Mitochondrial RNA processing in absence of tRNA punctuations in octocorals
Source: BMC Mol Biol. 2017 Jun 17;18:16. doi: 10.1186/s12867-017-0093-0 (PMC5474008; doi:10.1186/s12867-017-0093-0)
Supplement: Supplementary file 6 — Additional file 6. Antisense strand-specific RT-PCR. [file 12867_2017_93_MOESM6_ESM.pdf]

Additional file 6: Antisense strand-specific RT-PCR.

Gel image showing results of Antisense strand-specific RT-PCR.

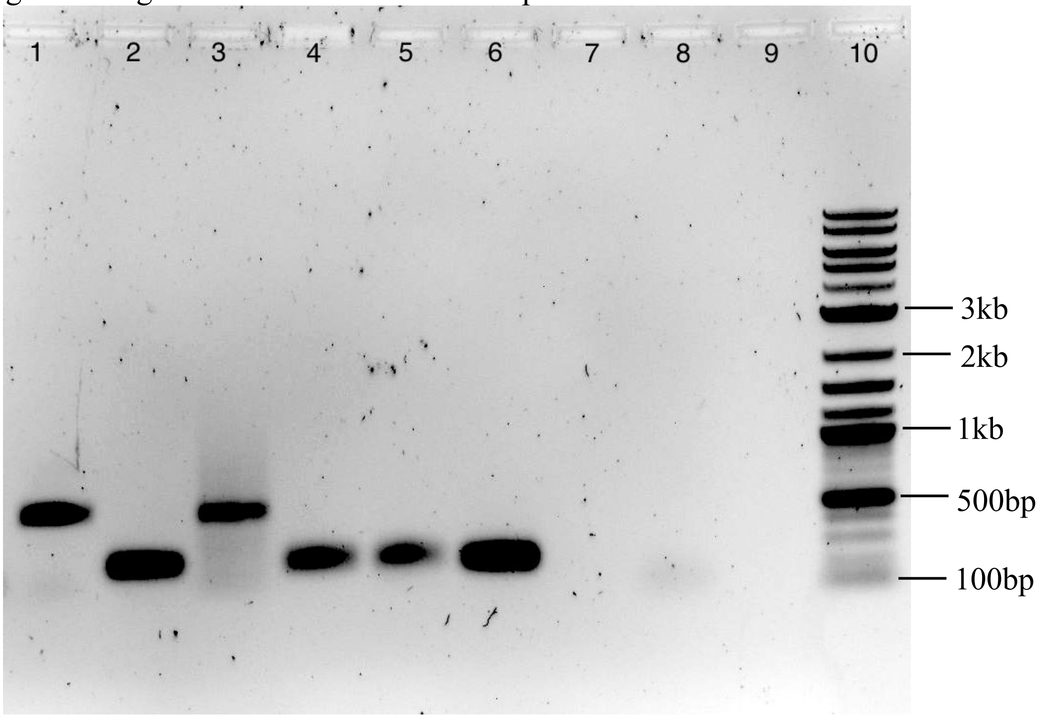

Gel Image details:

Lane	cDNA synthesis primer	RT-PCR primer pair
1.	Oligo(dT)	AAR2-SAF
2.	Oligo(dT)	AAR1-SAF
3.	AAR2	AAR2-SAF
4.	AAR2	AAR1-SAF
5.	AAR1	AAR1-SAF
6.	SAF	AAR1-SAF
7.	Blank	-
8.	Negative Control	AAR2-SAF
9.	Negative Control	AAR1-SAF
10.	2Log DNA ladder (NEB)	-

Primer details:
AAR1 5'-TTACTCCTACTGCCCATATTG-3'
AAR2 5'-TGTAGTTCGGATAATTGGGGG-3'
SAF 5'-TTAGCAGCCAATCGAACACC-3'
