# Supplementary material for: Mitochondrial RNA processing in absence of tRNA punctuations in octocorals
Source: BMC Mol Biol. 2017 Jun 17;18:16. doi: 10.1186/s12867-017-0093-0 (PMC5474008; doi:10.1186/s12867-017-0093-0)

Additional file 7: Stem-loop structures and conserved motif of IGRs between detected transcriptional units from *S.* cf. *cruciata*.

(A) Stem-loop structures of IGRs

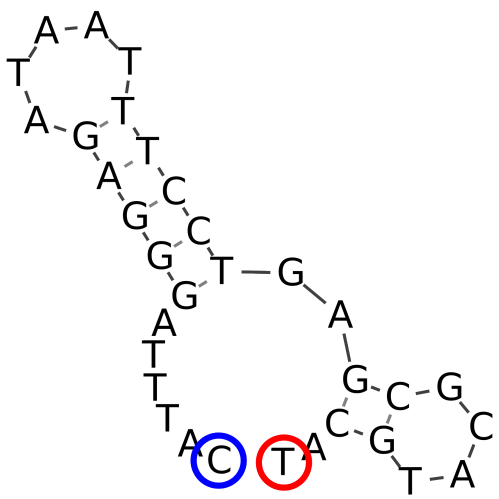

ND1-CytB IGR

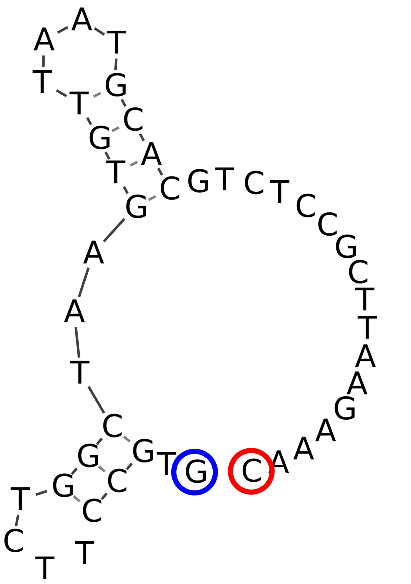

ND6-ND3 IGR

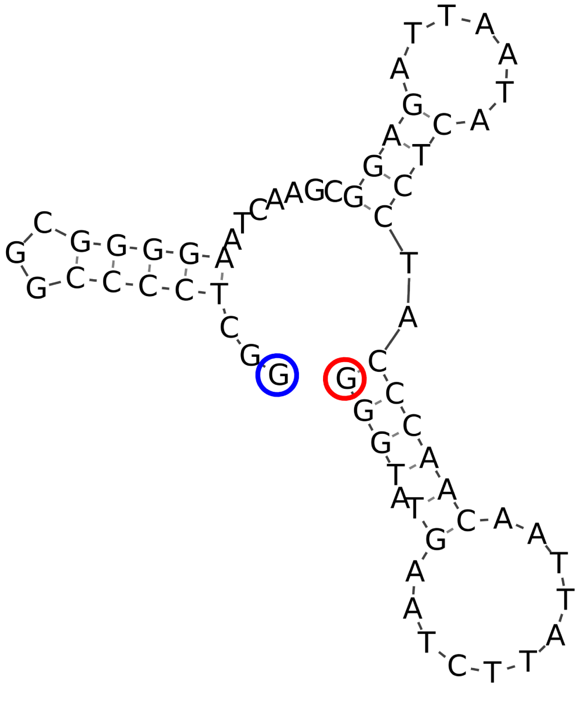

COIII-ATP6 IGR

(B) IGRs were aligned using GLAM2 from MEME Suite 4.11.2 (meme-suite.org/)

NAME	START	SITES	END	STRAND
COIII-ATP6	28	tctccgcttga	18	-
ND1-CytB	12	tctcc.ctaaa	3	-
ND6-ND3	29	tctccgcttaa	39	+

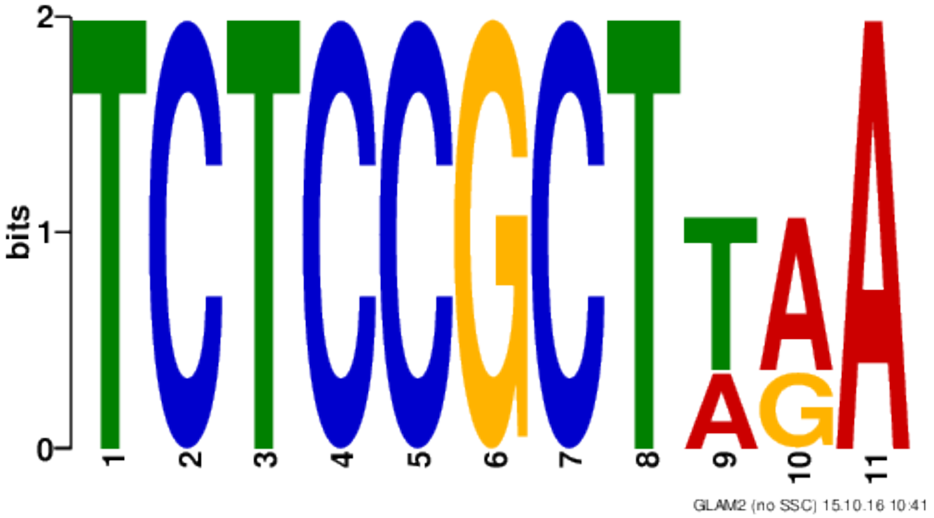

Supplement: Supplementary file 7 — Additional file 7. Stem-loop structures and conserved motif of IGRs between detected transcriptional units from S. cf. cruciata. [file 12867_2017_93_MOESM7_ESM.pdf]
